# Pityriasis Versicolor Resistant to Antifungal Drugs in a Patient in Lomé (Togo)

**DOI:** 10.1155/2022/5404913

**Published:** 2022-08-22

**Authors:** Julienne Teclessou, Koussake Kombate, Bayaki Saka, Séfako Abla Akakpo, Palokinam Pitche

**Affiliations:** ^1^Department of, Dermatology and STDs, University Teaching Hospital of Campus, Togo; ^2^Department of Dermatology and STDs, University Teaching Hospital of Sylvanus Olympio, Togo

## Abstract

**Background:**

Pityriasis versicolor (PV) is a ubiquitous superficial skin mycosis that often affects young adults. It is often effectively treated with local or oral antifungal agents. Cases of PV resistance to antifungal agents have been reported rarely. We report a case of antifungal resistant PV. *Observation*. A 22-year-old patient was followed since the age of 17 years in a dermatology outpatient clinic for hyperpigmented scaly macular lesions of the trunk and upper limbs. The clinical diagnosis of PV was retained. The patient was treated by fluconazole 300 mg/week before being lost to follow-up. He was seen again in 2019 (about 2 years later) for the same symptomatology and treated again by fluconazole and ciclopirox olamine cream without improvement. He was again lost to follow-up and seen again six months later. A mycological sample was taken and *Aspergillus niger* was isolated. The patient was treated by itraconazole for 6 weeks. The evolution was marked by a clinical status quo. The patient was again put on salicylated petroleum jelly 10% associated with terbinafine cream and then lost to follow-up.

**Conclusion:**

The emergence of fungal resistance to antifungal drugs does not spare PV. It can therefore be resistant to several antifungal drugs, leaving clinicians and patients in despair.

## 1. Introduction

Pityriasis versicolor (PV) is a superficial skin infection caused by fungi. It is a common ubiquitous dermatosis affecting young adults mainly in tropical regions with prevalence up to 40% [1]. PV manifests as well-limited scaly macules that may be separated by spaces of healthy skin or coalesce into irregular patches [2]. The lesions may be hypopigmented or hyperpigmented and are located preferentially in seborrheic areas. Lesions usually predominate on the back [2]. The diagnosis of PV is easy, essentially clinical. Wood's light examination is a diagnostic tool but is positive in only one-third of cases [3]. Fungal elements (filament spores) can be demonstrated by microscopic examination.

Therapeutically, the treatment of PV is based on topical or oral azole (imidazole) antifungals. They can be combined with azole-based antifungal foaming gels (ketoconazole foaming gel) [4]. However, recurrences are frequently described after a well-managed treatment. The recurrence rate is estimated at 60% after one year and can reach 80% in the second year [5]. In general, the resistance of mycoses to antifungal agents has become a major concern in clinical practice over the last 30 years. This resistance in fungal species is unevenly distributed worldwide and involves several fungal species [6]. In Africa, cases of resistance of candida species have been reported [7].

PV, apart from recurrences, can be resistant to antifungal treatments that have already been proven to manage the condition. Helou et al. in 2014 had reported a case of PV resistant to antifungal drugs [8]. We report a clinical case.

## 2. Observation

**Figure 1:**
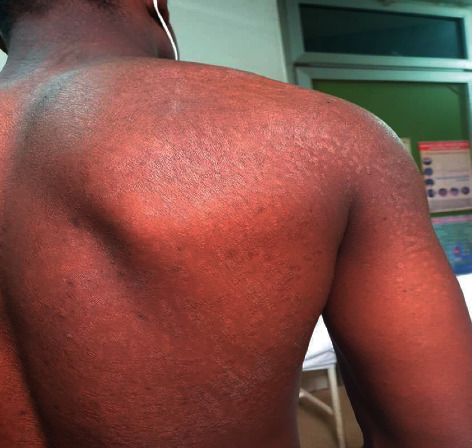
Scaly hypopigmented macular lesions of the trunk (months after the start of the second treatment).

**Figure 2:**
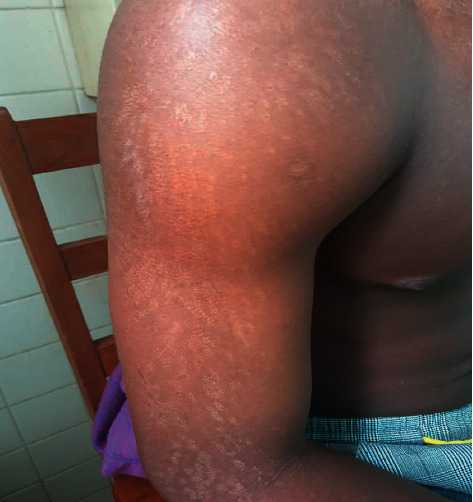
Pityriasis versicolor (six weeks after itraconazole treatment).

This is a 22-year-old patient, followed since the age of 17 years in the dermatology department of the Campus Hospital of Lomé for PV. The onset dates back to 2017 when the patient was seen on an outpatient basis for scaly hyperpigmented macular lesions with a positive chip sign. The lesions were located on the trunk and upper extremities and had been evolving for one year. There was no pruritus or pain. The clinical diagnosis of PV was retained and patient was treated by fluconazole 300 mg/week associated with ketoconazole foaming gel. The patient was then lost to follow-up. He was seen again two years later in October 2019 for the same symptoms. The interrogation allowed concluding that there was a discreet improvement of the lesions after the beginning of the treatment prescribed 2 years earlier followed by an aggravation. The treatment with fluconazole 300 mg/day was continued for 4 weeks and then replaced by ciclopirox olamine cream. The patient was seen once a month for 3 months without any improvement. He was again lost to follow-up. Six months later, the patient returned with the same symptoms (Figure 1). A mycological sample examination by microscopic in KOH identified a hyphae of *Aspergillus niger*. For technical reasons, the antifungigram (antifungal data) was not performed. Blood count, glycemia, and liver enzymes were normal. HIV serology was negative. The patient was treated by itraconazole 200 mg daily for 6 weeks. The evolution was marked by a clinical status quo of the lesions with persistence of scaly macules, the chip sign being always positive (Figure 2). The patient was then put on terbinafine cream and salicylated ointment 10%, in the course of treatment. The patient is lost again for more than 1 year.

## 3. Discussion

Two particularities are noted in this case: first, the persistence of lesions after a clinical diagnosis and a treatment by azoles; and secondly, the persistence of lesions after a mycological sampling and a treatment of isolated fungal species.

PV is a common dermatosis with an easy diagnosis based mainly on clinical findings. This clinical diagnosis can be supplemented by sampling of the scales (by Scotch or curette scraping) for mycological examination to identify the responsible fungal species. In our case, the diagnosis was based on clinical signs. However, given the persistence of the lesions after several well-conducted antifungal treatments, a direct microscopy examination in KOH was performed to identify the causal agent. Wood's light is also a diagnostic feature of the condition. However, it is not positive in all cases [8]. In our case, Wood's light examination was not performed for technical reasons.

PV can be managed by both general practitioners and dermatologists. Treatment is based on topical or oral antifungal agents. Most systemic or topical antifungal agents used in the treatment of PV are effective according to the study by Hu and Bigby [9]. Thus, topical fungistatic or fungicidal azoles are often used successfully in the treatment of PV [3]. In our case, these antifungals were also used unsuccessfully. Although our patient's follow-up was interrupted several times by periods of loss to sight, each time we started treatment we always had a sufficient follow-up period to assess the efficacy of the treatment.

Oral ketoconazole, the first available oral treatment for PV with multiple regimens [3], was the first-line treatment in our patient. It was offered at a regimen of 300 mg per week, close to the 400 mg per week regimen [10]. Also, itraconazole is an effective treatment with low toxicity used in the treatment of PV [11–13]. It is a triazole that has a broad spectrum of activity compared to fluconazole [6]. In contrast to fluconazole, itraconazole has been shown to be effective in fungal infections caused by *Aspergillus niger* [6]. In the absence of a scotch test performed at the start of treatment in our patient, we treated with fluconazole. But, the isolation of the *Aspergillus* strain prompted the initiation of itraconazole. We did not notice any improvement in the patient after the start of itraconazole. This leads us to conclude that there is resistance to the treatment. Few cases of PV resistance to antifungal drugs have been reported in the literature. Helou et al. in 2014 reported a similar case of PV resistant to antifungal drugs [8]. Several similarities can be noted with our case, notably the use of fluconazole from the first consultation, the persistence of the lesions, and then the use of itraconazole.

In our case, as in Helou et al.'s case, we were unable to assess the effect of salicylated petroleum jelly on the lesions as our patient was again lost to follow-up.

This observation again shows an increasing increase in antifungal resistance to the increasingly described fungal species [14]. Other antifungal agents such as Rilopirox (a synthetic pyridine derivative) and lanoconazole (an azole) known to be effective in the management of PV [5] were not prescribed in our patient due to their availability.

Resistant PV also poses a psychological problem for patients. Indeed, the unsightly appearance of the lesions could be a source of social rejection for the patient, especially when he is an adolescent or young adult. Our patient, aged 22, was seen several times in the department despite repeated therapeutic failure and periods of loss of sight. This could be explained by the unsightly aspect of the lesions and the permanent quest for healing.

## 4. Conclusion

Pityriasis versicolor is a ubiquitous superficial cutaneous mycosis with an easy diagnosis based essentially on clinical findings. Most cases of PV respond to conventional antifungal treatments. However, the emergence of fungal resistance to antifungal agents does not spare PV. Thus, cases of PV resistant to several antifungals may be noted and cause despair to clinicians and patients.
